# Supplementary material for: Florigen governs shoot regeneration
Source: Sci Rep. 2021 Jul 1;11:13710. doi: 10.1038/s41598-021-93180-1 (PMC8249374; doi:10.1038/s41598-021-93180-1)
Supplement: Supplementary file 1 — Supplementary Information. [file 41598_2021_93180_MOESM1_ESM.pdf]

Florigen governs shoot regeneration.

Yaarit Kutsher, Michal Fisler, Adi Faigenboim, Moshe Reuveni

Supplemental material

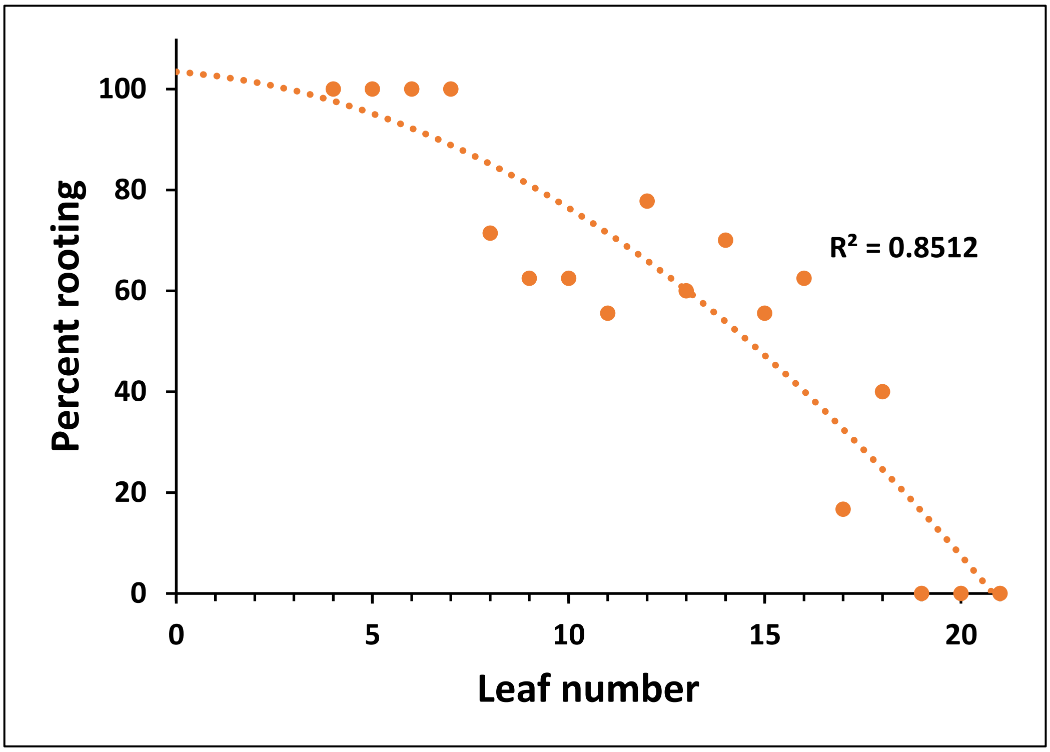

Figure S1: Percent rooting of tobacco leaves. Tobacco plants were grown in pots, and leaves were detached from the stem with the petioles. The leaves petioles were placed in agar containing one mg/L IBA with 2% sucrose. Rooting was calculated as the number of leaves that produced roots from a batch of leaves at the same location on the stem. Original rooting is shown in figure 1.

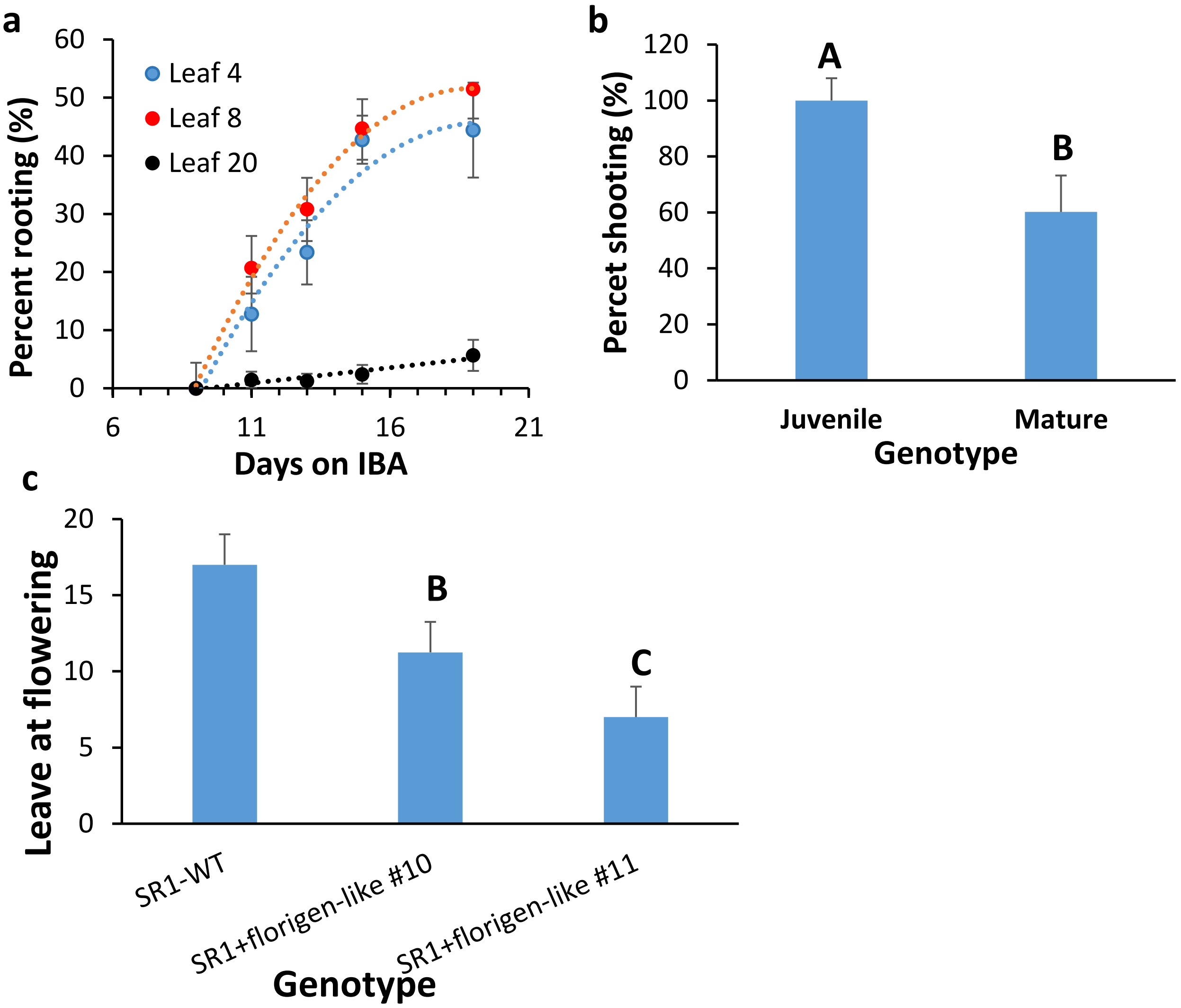

Figure S2: Percent shooting **(a)** or rooting **(b)** of tobacco leaf segments. Tobacco plants were grown in pots, and leaves were detached from the stem and cut into segments. The leaf segments were placed on Reg medium agar containing 3% sucrose for shooting and agar containing one mg/L IBA for rooting. Shooting or Rooting percent was calculated as the number of segments that produced shoots or roots from a batch of leaf segments on the same plate. **c**: Avocado floeigen induces early flowering when expressed in tobacco plants.

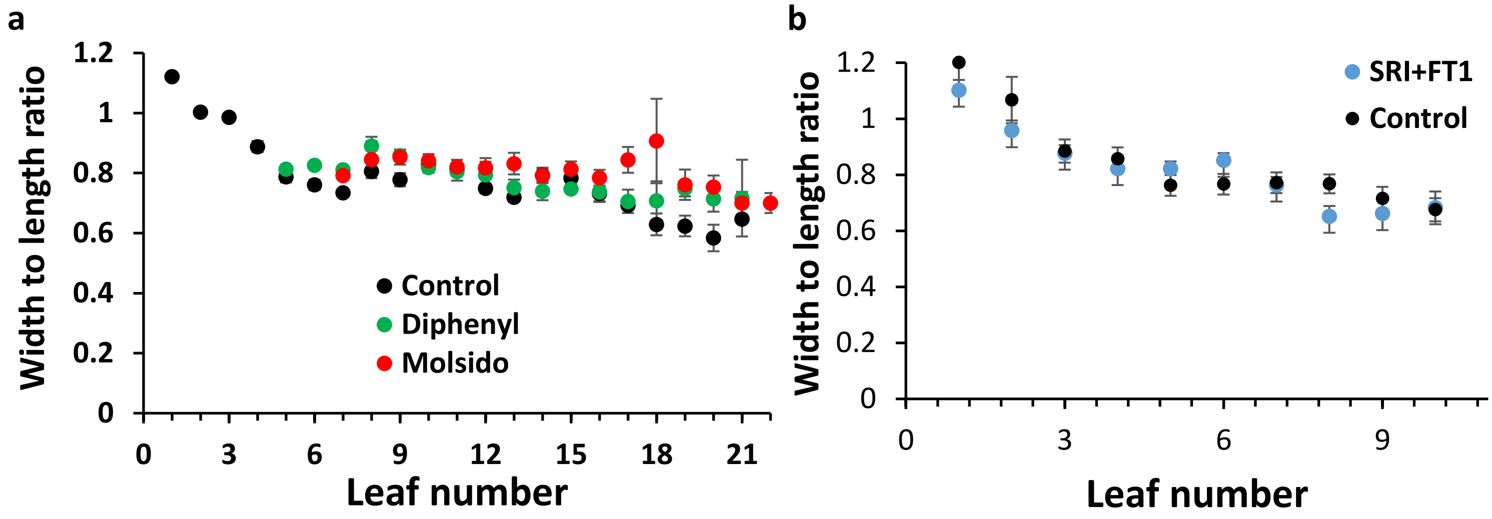

Figure S3: Width to length ratio of tobacco leaves. **a**: Tobacco plants were grown from seed in pots and sprayed at the 7 to 8 leaf stage with either water or Diphenyl (five μM) or Molsido (five μM) solutions. The leaves' length and width were measured at flowering, and the ratio width to length was calculated. **b:** SR1 (Control) or SR1 transformed with FT1 (FT1) tobacco plants were grown from seed in pots and leaves' length and width were measured at flowering for FT1 and at the same age in SR1, the ratio width to length was calculated. Each point is the mean $\pm$ se of at least five individual plants.

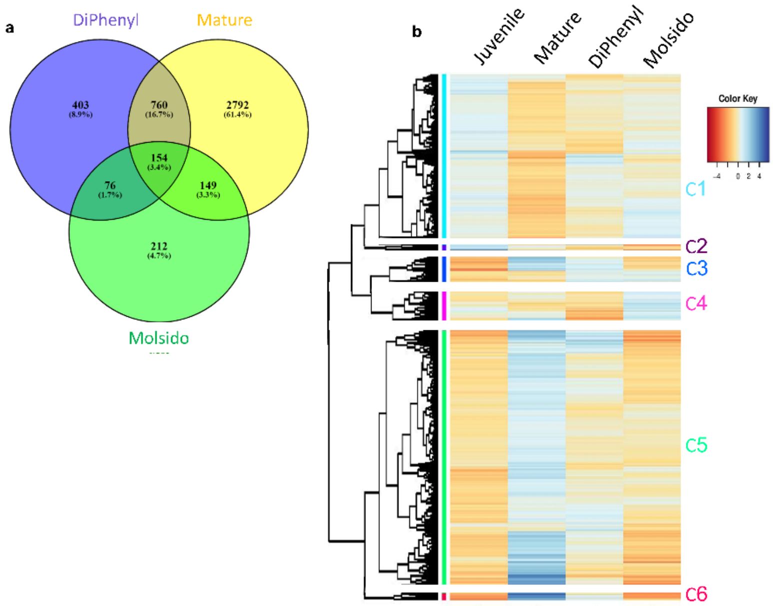

Fig. S4: Differential gene expression between juvenile and mature leaves and juvenile leaf treated with DiPhenyl and juvenile leaf treated with Molsido **a**: Number of mRNAs expressed differently than the juvenile samples. Plants grew on an agar medium supplemented with 2% sucrose and 5µM DiPhenyl or 5µM Molsido. At the seven-leaf stage, leaf blade disks were cut and RNA extracted and sent to RNA-seq. The experiment was done in two replicates with five plants each. **b**: Visualisation by heatmap of genes clusters whose mRNA expression increase or decreases at the juvenile, mature, juvenile leaf treated with DiPhenyl and juvenile leaf treated with Molsido.

Table S1: Expression level of possible *TEMPRANILLO* gene in tobacco juvenile or mature leaves or juvenile leaves from plants treated with Diphenyl (5 μM) or Molsido (5 μM) after germination.

Gene ID	Gene description	Juvenile expression level±SD	Juvenile +Diphenyl expression level±SD	Mature expression level±SD	Juvenile +Molsido expression level±SD
Nitab4.5_0000188g0320	B3 domain-containing protein At3g18960 IPR003340 Transcriptional factor B3	13.0±0.0	9.1±0.6	12.3±0.0	11.8±0.0
Nitab4.5_0000316g0140	B3 domain-containing protein At1g20600 IPR003340 Transcriptional factor B3	0.0±0.0	0.0±0.0	0.0±0.0	0.0±0.0
Nitab4.5_0000380g0010	B3 domain-containing protein Os03g0212300 IPR003340 Transcriptional factor B3	9.2±2.4	13.2±0.7	12.9±3.8	12.2±1.3
Nitab4.5_0000519g0250	AP2 domain-containing transcription factor IPR003340 Transcriptional factor B3	0.0±0.1	0.0±0.1	0.0±0.0	0.0±0.1
Nitab4.5_0000573g0030	Auxin response factor 24 IPR003340 Transcriptional factor B3	6.8±0.8	23.1±9.6	15.6±2.0	6.2±0.3
Nitab4.5_0000733g0200	B3 domain-containing protein At3g18960 IPR003340 Transcriptional factor B3	9.1±1.9	8.0±1.2	9.8±0.5	10.0±0.4
Nitab4.5_0000799g0050	Auxin response factor 24 IPR003340 Transcriptional factor B3	10.4±1.5	18.1±6.5	25.8±4.3	18.7±1.7
Nitab4.5_0000813g0020	B3 domain-containing transcription factor ABI3 IPR003340 Transcriptional factor B3	0.0±0.0	0.0±0.0	0.0±0.0	0.0±0.0
Nitab4.5_0001034g0090	B3 domain-containing protein Os01g0234100 IPR003340 Transcriptional factor B3	6.0±0.2	6.1±0.7	8.5±0.1	7.1±0.3
Nitab4.5_0001101g0130	B3 domain-containing protein At5g42700 IPR003340 Transcriptional factor B3	0.8±0.8	0.1±0.1	0.4±0.4	0.6±0.6
Nitab4.5_0001657g0040	B3 domain-containing transcription factor ABI3 IPR003340 Transcriptional factor B3	0.0±0.0	0.0±0.0	0.0±0.0	0.0±0.0

Nitab4.5_0001663g0030	B3 domain-containing protein At3g18960 IPR003340 Transcriptional factor B3	3.3±0.1	2.2±0.6	2.8±0.6	3.0±0.8
Nitab4.5_0001888g0040	B3 domain-containing protein At1g20600 IPR003340 Transcriptional factor B3	0.0±0.0	0.0±0.0	0.0±0.0	0.0±0.0
Nitab4.5_0002172g0100	AP2 domain-containing transcription factor IPR003340 Transcriptional factor B3	0.0±0.0	0.0±0.0	0.0±0.0	0.0±0.0
Nitab4.5_0003194g0040	B3 domain-containing protein Os05g0481400 IPR003340 Transcriptional factor B3	0.3±0.1	0.6±0.4	0.7±0.5	0.5±0.2
Nitab4.5_0000351g0250	NtFT1	0.0±0.0	3.6±1.9	2.1±1.2	0.0±0.0

Table S2: Expression level of possible *SQUAMOSA PROMOTER BINDING-LIKE* gene in tobacco juvenile or mature leaves or juvenile leaves from plants treated with Diphenyl (5 μM) or Molsido (5 μM) after germination.

Gene ID	Gene description	Juvenile expression level±SD	Diphenyl expression level±SD	Mature expression level±SD	Molsido expression level±SD
Nitab4.5_0000016g0300	Squamosa promoter-binding protein IPR004333 Transcription factor, SBP-box	3.4±0.3	3.1±0.1	4.9±1.2	4.6±0.1
Nitab4.5_0000059g0380	Squamosa promoter binding protein-like 1 IPR004333 Transcription factor, SBP-box	10.6±2.2	8.5±1.2	11.5±1.4	10.7±0.3
Nitab4.5_0002994g0100	Squamosa promoter binding protein-like 1 IPR004333 Transcription factor, SBP-box	38.3±0.9	40.5±7.0	49.8±3.3	31.1±1.9
Nitab4.5_0000222g0290	Squamosa promoter binding protein-like 1 IPR004333 Transcription factor, SBP-box	62.6±6.6	64.2±14.6	82.2±6.6	50.0±3.0
Nitab4.5_0000225g0020	Squamosa promoter binding protein-like 1 IPR004333 Transcription factor, SBP-box	1.4±0.4	0.8±0.4	1.7±0.0	3.4±0.4
Nitab4.5_0000363g0110	Squamosa promoter binding protein-like 1 IPR004333 Transcription factor, SBP-box	10.0±0.8	7.4±1.0	11.1±0.7	12.1±1.2
Nitab4.5_0000700g0020	Squamosa promoter binding protein-like 1 IPR004333 Transcription factor, SBP-box	7.4±0.7	6.4±0.5	7.5±1.6	7.5±0.1
Nitab4.5_0000745g0150	Squamosa promoter binding protein-like 1 IPR004333 Transcription factor, SBP-box	29.4±2.2	18.2±4.1	50.1±2.3	28.9±2.0
Nitab4.5_0000991g0020	Squamosa promoter-binding protein IPR004333 Transcription factor, SBP-box	0.0±0.0	0.1±0.1	0.6±0.6	0.2±0.1
Nitab4.5_0000638g0040	Squamosa promoter binding-like protein IPR004333 Transcription factor, SBP-box	1.2±0.1	1.1±0.5	1.8±0.5	2.2±0.3
Nitab4.5_0000210g0190	Squamosa promoter binding-like protein IPR017238 Squamosa promoter-binding protein	0.2±0.0	0.6±0.4	0.8±0.5	0.0±0.0

Nitab4.5_0002219g0060	Squamosa promoter binding-like protein IPR004333 Transcription factor, SBP-box	3.2±1.0	0.6±0.6	8.6±2.8	6.0±1.0
Nitab4.5_0002299g0030	Squamosa promoter-binding protein IPR004333 Transcription factor, SBP-box	3.2±0.1	13.7±8.2	43.5±6.8	5.7±0.4
Nitab4.5_0000210g0160	Squamosa promoter binding protein 1 IPR004333 Transcription factor, SBP-box	1.8±0.8	5.8±2.3	12.8±3.2	2.5±0.2
Nitab4.5_0001752g0040	Squamosa promoter binding-like protein IPR004333 Transcription factor, SBP-box	1.0±0.1	0.5±0.3	2.1±0.2	1.7±0.2
Nitab4.5_0003348g0050	Squamosa promoter binding-like protein IPR017238 Squamosa promoter-binding protein	7.0±0.3	12.7±5.5	18.8±1.3	10.9±2.3
Nitab4.5_0004959g0040	Squamosa promoter binding-like protein IPR004333 Transcription factor, SBP-box	0.0±0.0	0.2±0.0	0.1±0.0	0.2±0.2
Nitab4.5_0001315g0110	Squamosa promoter-binding-like protein 11 IPR004333 Transcription factor, SBP-box	0.8±0.1	0.0±0.0	0.1±0.1	0.7±0.1
Nitab4.5_0000067g0130	Squamosa promoter-binding-like protein 11 IPR004333 Transcription factor, SBP-box	24.6±4.7	20.0±0.4	12.7±3.2	27.5±3.5
Nitab4.5_0000861g0050	Squamosa promoter-binding-like protein 11 IPR004333 Transcription factor, SBP-box	7.1±2.3	1.7±0.1	5.7±1.3	9.0±0.4
Nitab4.5_0003900g0020	Squamosa promoter-binding-like protein 11 IPR004333 Transcription factor, SBP-box	5.0±0.5	3.9±0.6	7.4±1.6	8.7±2.2
Nitab4.5_0000027g0060	Squamosa promoter binding protein 3 IPR004333 Transcription factor, SBP-box	4.7±1.0	5.9±1.8	19.2±3.6	5.4±0.7
Nitab4.5_0000509g0030	Squamosa promoter binding protein 3 IPR004333 Transcription factor, SBP-box	3.0±1.2	1.5±0.6	1.0±0.1	0.6±0.1
Nitab4.5_0000509g0040	Squamosa promoter binding protein 3 IPR004333 Transcription factor, SBP-box	1.6±0.3	0.8±0.8	0.7±0.1	0.8±0.2

Nitab4.5_0001118g0090	Squamosa promoter binding protein 3 IPR004333 Transcription factor, SBP-box	0.8±0.3	0.8±0.1	0.5±0.2	0.7±0.2
Nitab4.5_0001328g0090	Squamosa promoter binding protein 3 IPR004333 Transcription factor, SBP-box	6.2±0.2	4.5±0.8	1.3±0.3	4.4±0.6
Nitab4.5_0001328g0100	Squamosa promoter binding protein 3 IPR004333 Transcription factor, SBP-box	1.2±0.6	0.8±0.6	0.8±0.4	2.1±0.1
Nitab4.5_0001797g0070	Squamosa promoter binding protein 3 IPR004333 Transcription factor, SBP-box	4.7±1.7	4.7±3.1	1.4±0.3	9.8±1.6
Nitab4.5_0002467g0020	Squamosa promoter binding protein 3 IPR004333 Transcription factor, SBP-box	4.3±1.5	5.8±1.4	13.1±0.5	8.0±1.0
Nitab4.5_0000351g0250	NtFT1	0.0±0.0	3.6±1.9	2.1±1.2	0.0±0.0
